# Notes on Cases Treated by Electricity

**Published:** 1873-08

**Authors:** G. W. Friou

**Affiliations:** Surgeon to Southern Dispensary, Brooklyn N. Y.


ART. III.—*Notes on Cases Treated by Electricity.* By G. W. Friou, M. D., Surgeon to Southern Dispensary, Brooklyn N. Y.

For several years I have taken up the subject of medical electricity, and studied its wonderful therapeutics with much pleasure, and by your kind invitation will contiuue the subject by submitting a few desultory notes of cases treated during the past year and one while practicing in New Orleans

Mrs. C., age 33 years, tall, very slender in appearance, is the mother of seven children, was married in her sixteenth year, has enjoyed excellent health up to two years ago, when over mental excitement (setting up with a child for weeks which died) left her in tremors, dependent upon a weakening of the muscular and nervous system, so much so as to prevent her doing anything requiring steady fingers; she could not even read, unless an assistant held the book. Had been under the treatment of an experienced medical gentlemen for fifteen months when, with his consent, she applied to be treated with electricity. The first application of half an hour was July 1st, after which there was much improvement. They were continued at intervals for a month, when she began to take two a week regularly up to September 15th. About the first of August, noticing a large Goitre (Bronchocele) of seventeen inches circumference, I suggested that we try to destroy it with the Faradic machine. I was skeptical of results but noticing that Drs. Beard and Rockwell cited cases of recoveries and also Dr. Meyers in his work mentions various kinds of tumors cured, thought we would try. My method was the one recommended by Dr. Meyers. At the time she ceased the use of the machine the Goitre was reduced to twelve inches and scarcely noticable, while her general health is excellent, having become quite fleshy and entirely freed from her old malady.

Mrs. N., age 28 years. She is of small frame. I found her suffering from all the symptoms of Hysterical dyspepsia. Had been under the treatment of one of the most distinguished of our profession who had tried the usual remedies. I prescribed for her a mixture of Pepsine and Bismuth. She took her first application September 7th. I used Dr. Kidder's machine and gave her a general Faradisation. After several applications with rapid improvement

I suggested as auxilary to the treatment the use of dumb bells. At her next visit she stated that being so much improved she had decided to dismiss half of her domestics and would substitute the broom for dumb hells. She had in all eight applications, and was dismissed entirely cured.

In the spring of 1869 I was called to see Mr. P., age 27 years, who fell from the roof of his manufactory, a distance of fourteen feet, and struck upon his back near or about the first lumbar vertebra. I saw him several hours after, when I found complete paraplegia. On examination I could not detect any external laceration or fracture, but the parts were a little tumid. I ordered cups to spine. After they were taken off, a poultice and sedative mixture for the night. But early in the morning I was called in haste to find him suffering from distended bladder and considerable tympanitis. I catheterized and ordered a calomel purge. Saw him again in the evening; no better. I again used the catheter, and filled his bowels with warm soap-suds. March 6th, still no better. Continued treatment with stimulating embracations and blisters to spine. March 7th, found one of his friends, an old woman, had put him into a large tub of scalding water. When seen the entire cutis was removed from the scrotum and its surroundings, exposing the sphincter muscle. Although the entire seat was sloughing, he had no sense of feeling more than an icy coldness. I used simple dressings and ordered one of Dr. Kidder's Faradic machines and began the treatment March 7th. I visited him twice a day for a few weeks, and was necessitated to assist nature by the use of catheter and pump syringe daily. The sloughing gradually subsided under the use of the machine, in a few days he began to experience sensation when the negative electrode was applied to the extremities. The treatment was continued three months when he was able to walk about with two sticks (he could not use crutches) with a peculiar dragging motion, and before the close of the year he had recovered the use of all his functions. A little over two years from the time of the accident his wife gave birth to a son.

